# The *Ginkgo biloba* Extract EGb 761 Modulates Proteasome Activity and Polyglutamine Protein Aggregation

**DOI:** 10.1155/2014/940186

**Published:** 2014-06-05

**Authors:** Marcel Stark, Christian Behl

**Affiliations:** Institute for Pathobiochemistry, University Medical Center of the Johannes Gutenberg University, Duesbergweg 6, 55099 Mainz, Germany

## Abstract

The standardized *Ginkgo biloba* extract EGb 761 has well-described antioxidative activities and effects on different cytoprotective signaling pathways. Consequently, a potential use of EGb 761 in neurodegenerative diseases has been proposed. A common characteristic feature of a variety of such disorders is the pathologic formation of protein aggregates, suggesting a crucial role for protein homeostasis. In this study, we show that EGb 761 increased the catalytic activity of the proteasome and enhanced protein degradation in cultured cells. We further investigated this effect in a cellular model of Huntington's disease (HD) by employing cells expressing pathologic variants of a polyglutamine protein (polyQ protein). We show that EGb 761 affected these cells by (i) increasing proteasome activity and (ii) inducing a more efficient degradation of aggregation-prone proteins. These results demonstrate a novel activity of EGb 761 on protein aggregates by enhancing proteasomal protein degradation, suggesting a therapeutic use in neurodegenerative disorders with a disturbed protein homeostasis.

## 1. Introduction

The widely used standardized* Ginkgo biloba* extract EGb 761 is a multifaceted composition of pharmacologic effective substances, especially terpene trilactones (6%) and flavonol glycosides (24%), as well as a variety of unknown substances (about 13%) [1]. The main constituents of the flavonoid fraction are the antioxidants quercetin, kaempferol, and isorhamnetin [1]. Due to its antioxidant effects, EGb 761 has been used as a natural treatment for a variety of disorders associated with cellular oxidative stress, like cardiovascular and neurodegenerative diseases [2], including Alzheimer's disease (AD) [3, 4]. It was shown that in AD the treatment with EGb 761 provides protective effects through a combination of antioxidative [5], free radical scavenging [6], antiamyloidogenic [7], and antiapoptotic properties [8]. In addition, it was demonstrated that EGb 761 has beneficial properties by promoting the induction of protective phase 2 genes, mediated through the NRF2-KEAP1 signaling pathway [9, 10].

One common hallmark of neurodegenerative diseases, like AD and also Huntington's disease (HD), is the formation of aberrant protein aggregates [11]. For HD, its neuropathology is caused due to N-terminal CAG-repeat mutations in exon 1 of the* huntingtin* gene, leading to expansions of repeated glutamine (Q) residues in the encoded protein (polyQ protein) [12]. The expansion length of the polyQ protein is crucial for the accelerated formation of polyQ aggregates and associated aberrant cellular dysfunctions [13]. Misfolded proteins are being immediately removed through the proteasome or if their degradation fails, these proteins accumulate and form protein aggregates [14]. PolyQ aggregates assemble to insoluble inclusion bodies, containing amyloid-like fibers of polyQ proteins, numerous cytoplasmatic proteins, and proteins from the ubiquitin-proteasome system (UPS) [15, 16]. The withdrawal of proteins from the UPS decreases the efficiency in protein degradation, further causing a disturbed protein homeostasis [17]. In addition, aberrant monomeric and oligomeric expanded polyQ proteins can promote further pathologic cellular dysregulations and toxicity [18].

In the present study, we have examined the effects of EGb 761 on basal enzymatic activity of the proteasome and the associated proteasomal protein degradation. We further tested the impact of EGb 761 on the modulation of a proteasome impairment, occurring in cells expressing aberrantly expanded polyQ proteins. In fact, we could confirm the modulating effects of EGb 761 on proteasome activity even under these conditions. In this context, we further assessed the properties of EGb 761 on the formation of polyQ aggregates. We demonstrated that EGb 761 also modulated the accumulation of expanded polyQ proteins through a more efficient proteasomal degradation. Conclusively, these results indicate that EGb 761 modulates proteasome activity and alleviates the pathologic aggregation of polyQ proteins, suggesting novel potential therapeutic targets for EGb 761 for neurodegenerative diseases.

## 2. Materials and Methods

### 2.1. Materials

All materials were obtained from Sigma-Aldrich (Germany) or Invitrogen (Germany). Stock solutions of chemicals used in this study were prepared in DMSO. Different to standard materials, SUC-LLVY-AMC was purchased from Alexis and MG132 from Calbiochem. The standardized* Ginkgo biloba* leaf extract EGb 761 was provided by Dr. Willmar Schwabe Pharmaceuticals (Germany). EGb 761 extract used is a registered trademark of Dr. Willmar Schwabe Pharmaceuticals. Stock solutions of EGb 761 were prepared in DMSO with a concentration of 150 mg/mL EGb 761. DMSO with a final concentration of 0.1% was used as vehicle treatment.

### 2.2. Antibodies

All antibodies were obtained from commercial sources. Mouse-monoclonal anti-eGFP (1 : 1000) and mouse-monoclonal anti-Tubulin (1 : 3000) were obtained from Sigma-Aldrich (Germany). Rabbit-polyclonal anti-20S proteasome *α*1 (H-95) (1 : 1000) was purchased from Santa Cruz Biotechnology (USA). Rabbit-polyclonal anti-polyubiquitin (1 : 1000) was obtained from Dako (USA). Secondary antibodies (1 : 10000) were purchased from Jackson ImmunoResearch (USA).

### 2.3. Cell Culture, Transfections, and Microscopy

In this study, we used HEK293 (purchased from the American Type Culture Collection, USA) and d2GFP-HEK cells [19]. All cells were maintained in media of DMEM, supplemented with 10% [v/v] fetal bovine serum, 1% [v/v] pyruvate, 100 U/mL penicillin, and 100 *μ*g/mL streptomycin, and kept in a 5% CO_2_-humidified atmosphere at 37°C. During cultivation, medium was refreshed daily and cells were controlled for viability. Cells were treated 24 h after transfer or transfection with indicated concentrations of EGb 761 or 0.1% DMSO (vehicle). To achieve transient expression of variant polyQ fusion proteins, cells were transfected using electroporation technique with the Amaxa Nucleofector I (Lonza, Germany) [19]. For microscopic analysis of GFP fluorescent from polyQ aggregates, cells were fixed with ice-cold methanol and additionally stained with DAPI. Quantification of the amount and fluorescence intensity of polyQ aggregates was carried out with a conventional inverted Axiovert 200 (Zeiss, Germany) and analyzed by using ImageJ software (NIH, USA).

### 2.4. Plasmids for the Expression of PolyQ Proteins

To generate plasmids expressing polyQ fusion proteins, we used PCR-based DNA cloning with the In-Fusion technique (Clontech, USA). To get plasmids encoding for polyQ proteins with different length of glutamine repeats, we used DNA sequences with variant numbers of CAG repeats in the encoded N-terminal region of the huntingtin protein (exon 1) [13]. Therefore, we subcloned the coding sequences from the plasmids p426-25Q-GPD, p426-Q46-GPD, and p426-103Q-GPD (obtained from Addgene; described by Krobitsch and Lindquist [13]) into the GFP-N1 plasmids (Clontech, USA). For In-Fusion cloning, we used the following primer pairs: reverse primer htt_GFP 5′-TCT AGA GTC GCG GCC TTA CTT GTA CAG CTC GTC C-3′ and forward primer htt_GFP 5′-CGC GGG CCC GGG ATC ATG GCG ACC CTG GAA AAG-3′. The resulting fusion plasmids encoded for polyQ fusion proteins with 25 glutamines (htt_Q25), 46 glutamines (htt_Q46), and 103 glutamines (htt_Q103) repeats.

### 2.5. Immunoblotting and Filter Retardation Assay

Immunoblotting was carried out with total protein lysates, prepared from scraping cells in lysis buffer (2% SDS and protease inhibitor mix). Protein concentration was determined by BCA method (Pierce, Thermo Scientific, USA). Proteins were separated with SDS-PAGE, using Bis-Tris gels with MES running buffer, executed on a Mini Protean system (Bio-Rad, Germany). Gels were transferred by Western blotting to nitrocellulose membranes (Schleicher&Schuell, Whatman, USA). Following incubation with primary and secondary antibodies, proteins were detected by chemiluminescence reaction using Immobilon (Millipore, Germany) substrates and visualized with LAS-3000 dark box (Fujifilm, Japan). Intensities of protein bands were quantified by using ImageJ software (NIH, USA). Filter retardation assay was performed as previously described [20]. Total cell lysates were diluted in assay buffer (50 mM DTT, 2% SDS) and boiled for 7 min. Samples were applied on cellulose membrane (0.2 *μ*m) in a Minifold I system (Schleicher&Schuell, Whatman, USA) with weak vacuum, followed by additional washing steps. The membrane was dried through maintaining the vacuum and finally probed with anti-eGFP antibody overnight. PolyQ aggregates were detected and quantified according to immunoblot analysis.

### 2.6. Measurement of Proteasome Activity

Determination of proteasome activity was accomplished by measuring hydrolysis of the fluorogenic peptide SUC-LLVY-AMC as previously described [21]. Thus, cells were cultivated in 96-well clear bottom black dish in phenol-red free DMEM. After settlement, cells were treated for 24 h with EGb 761 or vehicle. Then, culture medium was replaced with medium containing 100 *μ*M SUC-LLVY-AMC with or without 10 *μ*M MG132. Probes were incubated for 60 min and then lysed with reporter lysis buffer (Promega, USA). Turnover of SUC-LLVY-AMC was determined by measuring AMC fluorescence using Victor3V Multilabel counter (Perkin Elmer, USA) at 460 nm emission. Specific proteasome activity was calculated as the difference between total activity and activity in the presence of MG132.

### 2.7. Degradation of d2GFP by Using GFP Fluorescence

Analysis of protein degradation through the proteasome was performed with a modified GFP-based proteasome reporter protein (d2GFP), bearing a destabilizing sequence to enhance degradation through the proteasome [22]. Therefore, HEK293 cells stable expressing d2GFP (d2GFP-HEK) [19] were cultivated in 96-well clear bottom black dish in phenol-red free DMEM. After settlement, cells were treated for 24 h with increasing concentrations of EGb 761 or vehicle. Baseline GFP fluorescence was measured with Victor3V Multilabel counter (Perkin Elmer, USA) to investigate protein degradation through the proteasome. To show the specific modulation of GFP fluorescence through proteasome activity, cells were additionally challenged for 2 h with 10 *μ*M proteasome inhibitor MG132 or 0.1% DMSO as vehicle. Then, fluorescence was measured again. Accumulation of d2GFP protein in cells with proteasome inhibition was displayed by an increased GFP fluorescence [22]. Further analysis of d2GFP protein degradation was performed by measuring fluorescence decay in the presence of protein biosynthesis inhibitor cycloheximide (CHX). Cells were treated for 24 h with 150 *μ*g/mL EGb 761 or vehicle, followed by a challenge with 500 nM CHX or 0.1% DMSO. GFP fluorescence was measured using a Victor3V Multilabel counter at 37°C for 150 min in 30 min intervals. To determine d2GFP protein degradation, fluorescence of CHX-treated cells was subtracted with fluorescence values from DMSO-treated cells. The decline of GFP fluorescence indicated protein degradation ruling out effects of d2GFP expression.

### 2.8. Quantitative Real-Time Reverse-Transcription PCR (qRT-PCR)

Quantitative analysis of transcriptional regulation from catalytic proteasome genes was performed with qRT-PCR. Total RNA from treated cells was extracted using NucleoSpin RNA II Kit (Macherey-Nagel, Germany) according to the manufacturer's instructions. Resulting mRNA (1 *μ*g/*μ*L) was probed with Omniscript RT Kit (Qiagen, Germany) to prepare cDNA. Final qRT-PCR was performed with ABsolute qRT-PCR SYBR Green Fluorescein Kit (Thermo Scientific, USA) on the iCycler (BioRad, Germany), using the following sense and antisense primer pairs (100 pmol) as previously described [23]:* actin* (rev: 5′ CAG GTC CAG ACG CAG GAT GGC ′3; for: 5′CTA CAA TGA GCT GCG TGT GGC ′3);* psmb5* (rev: 5′ CAT CTC TGT AGG TGG CTT GGT ′3; for: 5′ AGG TTC TGG CTC TGT GTA TGC ′3);* psmb6* (rev: 5′ CAA ACT GCA CGG CCA TGA TA ′3; for: 5′ GAG GCA TTC ACT CCA GAC TG ′3);* psmb7* (rev: 5′ ACA ACC ATC CCT TCA GTT GC ′3; for: 5′ TGC AAA GAG GGG ATA CAA GC ′3). The relative expression ratio R of target genes in treated cells compared to untreated cells was calculated, using the relative expression software tool (REST) [24]. Actin was used as housekeeping gene (reference gene).

### 2.9. Statistical Methods

Statistical significance was calculated by using Student's* t*-test. Results were referred to as statistically significant at *P* values <0.05. Results are expressed as mean ± standard deviation (SD).

## 3. Results

### 3.1. Modulatory Effects of EGb 761 on Basal Proteasome Activity

**Figure 1:**
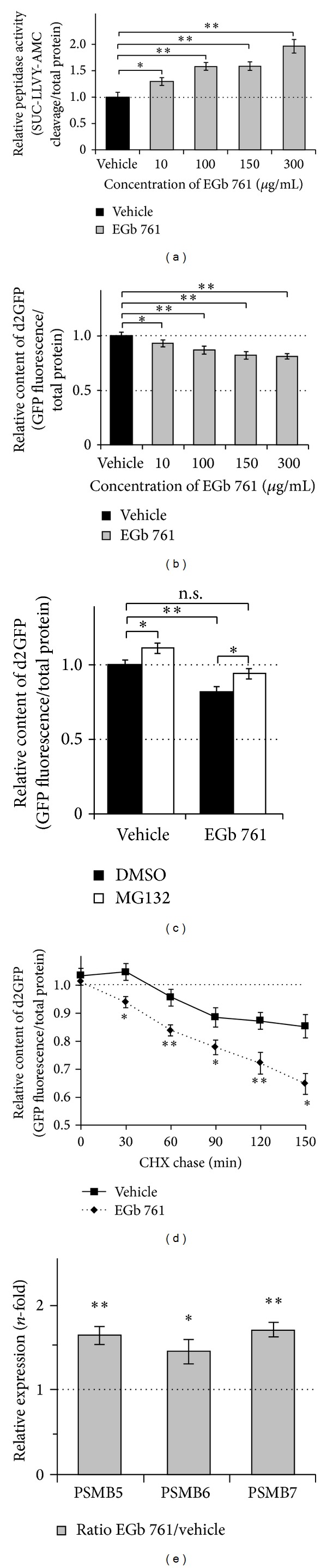
Effects of EGb 761 on basal proteasome activity. (a) HEK293 cells were treated for 24 h with indicated concentrations of EGb 761. Analysis of proteasomal peptidase (chymotrypsin-like) activity was assessed by the hydrolysis of SUC-LLVY-AMC in total cell lysates. Fluorescence of the cleaved AMC moiety was measured in the presence or absence of MG132 to achieve peptidase specificity. Values were adjusted to total protein content. Activity of vehicle-treated cells was arbitrarily set to 1; *n* = 4. (b–d) HEK293 cells with stable expressions of the proteasome reporter protein d2GFP (d2GFP-HEK) were treated for 24 h with indicated concentrations of EGb 761. Measurement of GFP fluorescence was used to investigate proteasomal degradation of d2GFP proteins. All achieved fluorescence intensities were finally adjusted to total protein content. (b) Cells were incubated with increasing concentrations of EGb 761 to investigate the specific, dose-depending effect on proteasome activity (a). Measurement of GFP fluorescence was used to assess the remaining d2GFP protein content as an indicator for an enhanced protein degradation by the proteasome. Values of vehicle-treated cells were arbitrarily set to 1. *n* = 5. (c) Previous assayed cells ((b); vehicle and 150 *μ*g/mL EGb 761 treatments) were additionally incubated for 2 h with the proteasome inhibitor MG132 or DMSO as control. The addition of MG132 led to an increase of fluorescence intensities in control and EGb 761-treated cells. Inhibition of proteasome activity showed the specific modulation of GFP fluorescence through proteasomal d2GFP degradation. Values of vehicle-treated cells without MG132 were arbitrarily set to 1. *n* = 4. (d) Cells were treated for 24 h with 150 *μ*g/mL EGb 761 or vehicle, followed by a chase with cycloheximide (CHX) to block synthesis of new d2GFP. Degradation kinetics of d2GFP was analyzed by measuring GFP fluorescence every 30 min. Fluorescence decay induced by CHX indicated the specificity of proteasomal d2GFP degradation. Values of each treatment at zero minutes were arbitrarily set to 1; *n* = 3. (e) HEK293 cells were treated for 2 h with 150 *μ*g/mL EGb 761 or vehicle and RNA was extracted for qRT-PCR analysis. Relative expression ratio of proteasome genes PSMB5, PSMB6, and PSMB7 in EGb 761-treated cells to vehicle-treated cells is shown; *n* = 3. (a–e) All values are reported as mean ± S.D. *P < 0.05 and **P < 0.01.

Several studies demonstrated that EGb 761 has various effects on intracellular signaling pathways, protein activities [25, 26], and protein aggregation [27]. Here, we investigated whether EGb 761 can also enhance proteasome activity. Therefore, we first tested the effects of EGb 761 on basal proteasome activity by investigating the proteasomal cleavage of SUC-LLVY-AMC by measuring the fluorescence of AMC [28]. Thus, we treated HEK293 cells with increasing concentrations of EGb 761 and measured the AMC fluorescence with or without the proteasome inhibitor MG132 as control. Our experiments revealed an EGb 761 dose-depending elevation of proteasome enzymatic activity up to 1.96-fold at 300 *μ*g/mL compared to control cells (Figure 1(a)) with an estimated and, based on our analysis, approximated EC_50_ value around 150 *μ*g/mL (1.59-fold to vehicle) (Figure 1(a)). In our cell system the application of EGb 761 up to a concentration of 300 *μ*g/mL did not affect cell viability (data not shown).

Next, we used HEK293 cells with a stable expression of the GFP-based proteasome reporter protein d2GFP (d2GFP-HEK) [19, 22] to analyze the effects of EGb 761 on proteasomal protein degradation. Therefore, we treated d2GFP-HEK cells with increasing concentrations of EGb 761 and measured the intensity of d2GFP fluorescence. We observed a decrease in d2GFP fluorescence with increasing concentrations of EGb 761 suggesting an increased degradation of d2GFP proteins with EGb 761 with a plateau phase at 150 *μ*g/mL (0.82-fold to vehicle) to 300 *μ*g/mL (0.81-fold to vehicle) (Figure 1(b)), assuming a saturation of protein degradation. Based on this data and the measured EC_50_ value (Figure 1(a)), we assigned 150 *μ*g/mL EGb 761 as an appropriate dosage for all further conducted experiments. To confirm the specificity of the enhanced d2GFP degradation by EGb 761 we subsequently chased the cells with the addition of 10 *μ*M MG132 for at least 2 h and measured the d2GFP fluorescence. Proteasome inhibition led to an increase of d2GFP fluorescence in vehicle-treated (1.11 fold) and EGb 761-treated cells (1.15-fold) (Figure 1(c)). Hereby, total levels of EGb 761-treated cells merely increased to levels of vehicle-treated cells without inhibitor (0.94-fold compared to DMSO) (Figure 1(c)), confirming that modulation of d2GFP fluorescence was mediated by proteasomal degradation (Figure 1(b)). To confirm these data, we tested the degradation of d2GFP protein in the presence of cycloheximide (CHX, 500 nM) in d2GFP-HEK cells with or without EGb 761. Here, we observed a general decay of d2GFP fluorescence with or without EGb 761, implicating the removal of d2GFP proteins and the absence of newly synthesized d2GFP proteins (Figure 1(d)). Furthermore, EGb 761 significantly accelerated the decay of d2GFP fluorescence compared to vehicle treatment starting 30 min after CHX incubation (Figure 1(d)), confirming an EGb 761-induced elevated proteasomal degradation of d2GFP.

As EGb 761 is a known inducer of phase 2 genes through the KEAP1-NRF2-ARE signaling pathway [10], we finally tested if EGb 761 is also capable of inducing the expression of the major proteasome genes (PSMB5, PSMB6, and PSMB7), harboring ARE-elements [29]. Analysis of cells treated for 2 h with EGb 761 by qRT-PCR revealed upregulated transcript levels of PSMB5 (1.65-fold), PSMB6 (1.45-fold), and PSMB7 (1.71-fold) to control cells (Figure 1(e)). Taken together, these results (Figures 1(a)–1(e)) suggest that an enhanced expression of proteasome genes by EGb 761 may result in an increased proteasome activity as shown also in other models [30].

### 3.2. EGb 761 Modulates Proteasome Activity in Cells Expressing PolyQ Proteins

**Figure 2:**
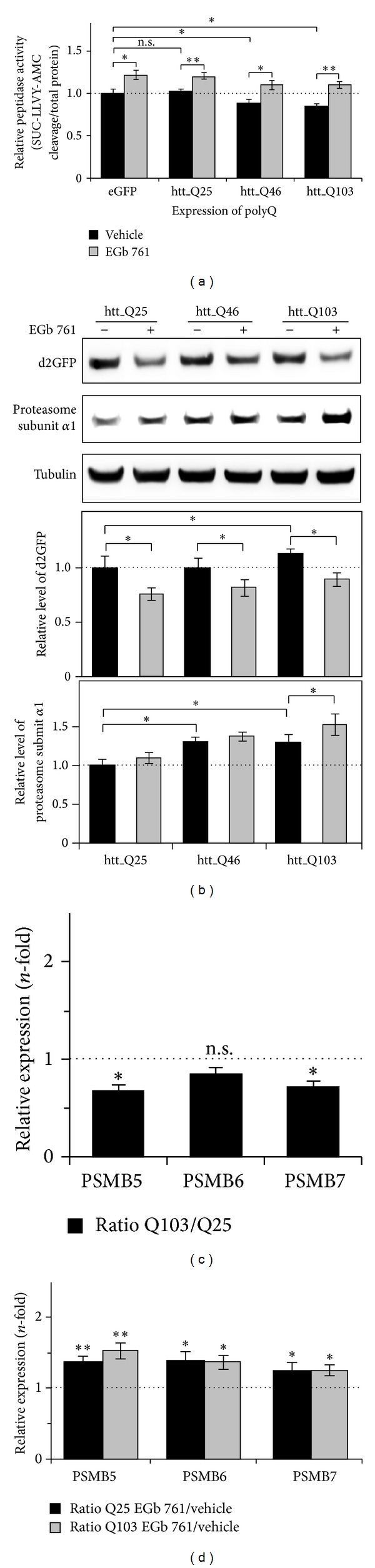
Modulation of proteasome activity by EGb 761 in cells expressing polyQ proteins. (a, c-d) HEK293 or (b) d2GFP-HEK cells were transiently transfected to express the polyQ fusion proteins with glutamine expansions of 25 (htt_Q25), 46 (htt_Q46), and 103 repeats (htt_Q103). Subsequent to settlement for 24 h cells were treated with 150 *μ*g/mL EGb 761 or vehicle for different time periods and further investigated. (a) HEK293 cells expressing eGFP and polyQ proteins (htt_Q25, Q46, and Q103) were treated for 24 h with EGb 761 or vehicle, followed by analysis of proteasomal peptidase activity in total cell lysates. Fluorescence of AMC moiety resulting from peptidase hydrolysis of SUC-LLVY-AMC was measured in the presence or absence of MG132 to achieve peptidase specificity. Values were adjusted to total protein content. Activity of vehicle-treated eGFP-expressing cells was arbitrarily set to 1; *n* = 3. (b) d2GFP-HEK cells expressing polyQ proteins (htt_Q25, Q46, and Q103) were treated for 24 h with EGb 761 or vehicle. Cell lysates of each sample were collected and analyzed by immunoblotting. Protein levels of d2GFP and proteasome subunit *α*1 were analyzed with their corresponding antibody to assess proteasome activity and status. Densitometric values of vehicle-treated and htt_Q25-expressing cells were arbitrarily set to 1; *n* = 4. (c-d) HEK293 cells expressing htt_Q25 and htt_Q103 for 24 h were analyzed by qRT-PCR for transcript levels of PSMB5, PSMB6, and PSMB7. (c) Quantification of basal expression ratio in cells expressing htt_Q103 to htt_Q25 (without EGb 761 treatment). (d) Analysis of cells additionally treated with EGb 761 or vehicle for 2 h. Expression ratio of proteasome genes in cells treated with EGb 761 compared to vehicle; *n* = 3. (a–d) All values are reported as mean ± S.D. *P < 0.05 and **P < 0.01.

The expression of expanded polyQ proteins is proposed to cause an impairment of proteasome activity [31]. We investigated whether EGb 761 treatment induces an enhanced proteasome activity in cells expressing polyQ proteins [13]. Therefore, HEK293 cells expressing eGFP or polyQ fusion proteins with different glutamine repeats (htt_Q25, htt_Q46, and htt_Q103) were treated with EGb 761 or vehicle and the proteasome activity was analyzed by using SUC-LLVY-AMC substrates. We did not find differences in proteasome activities between eGFP (arbitrarily set to 1-fold) and htt_Q25-expressing cells but a significant reduction in cells expressing htt_Q46 (0.88-fold) and htt_Q103 (0.84-fold) (Figure 2(a)), confirming that proteasome activity is impaired with the expression of expanded polyQ proteins [31]. Consistent with the observation of an EGb 761-induced increased proteasome activity in nontransfected normal HEK293 cells (Figure 1(a)), we observed a general EGb 761-induced increased proteasome activity in all cells, despite their protein expression (eGFP: 1.22-fold, htt_Q25: 1.20-fold, htt_Q46: 1.10-fold, and htt_Q103: 1.10-fold) (Figure 2(a)).

Next, we analyzed the effects of EGb 761 on d2GFP-HEK cells expressing htt_Q25, htt_Q46, and htt_Q103. Immunoblot analysis revealed no significant difference in d2GFP protein levels in vehicle-treated cells expressing htt_Q25 and htt_Q46 (Figure 2(b)), whereas htt_Q103-expressing cells showed elevated levels (1.13-fold to htt_Q25), indicating a lower degradation of d2GFP [31]. Accordingly, EGb 761 treatment significantly decreased d2GFP protein levels in all cells (htt_Q25: 0.76-fold, htt_Q46: 0.82-fold, and htt_Q103: 0.77-fold) to vehicle-treated cells (Figure 2(b)), suggesting a higher proteasome activity by EGb 761. The additional analysis of proteasome protein levels (20S proteasome subunit *α*1) revealed an unexpected increase of baseline levels in expressions of htt_Q46 (1.31-fold) and htt_Q103 (1.29-fold) to htt_Q25 (Figure 2(b)). Here, we speculate that this effect could result from the accumulation of active proteasome proteins with polyQ aggregates, further triggering the observed impairment of proteasome activity (Figure 2(a)). Nevertheless, EGb 761 treatment moderately increased proteasome protein levels in all cells that was clearly significant in htt_Q103-expressing cells (1.19-fold to vehicle) (Figure 2(b)) and indicating an EGb 761-induced expression of proteasome proteins (Figures 2(a) and 1(a)).

To further investigate the observed accumulation of proteasome proteins we analyzed the transcription of proteasome genes in cells expressing htt_Q25 and htt_Q103 by using qRT-PCR. Interestingly, analysis of cells expressing htt_Q103 exhibited a downregulation of proteasome genes (PSMB5: 0.66-fold; PSMB6: 0.85-fold; and PSMB7: 0.71-fold) compared to htt_Q25 (Figure 2(c)) confirming an inactivation of proteasome proteins with accumulation in polyQ aggregates. Conclusively, we investigated if the observed EGb 761-induced increase of proteasome protein levels resulted from higher expressions of proteasome genes. Analysis by qRT-PCR revealed that the treatment with EGb 761 significantly upregulated transcript levels of proteasome genes in cells expressing htt_Q25 (PSMB5: 1.36-fold; PSMB6: 1.38-fold; PSMB7: 1.24-fold) and htt_Q103 (PSMB5: 1.53-fold; PSMB6: 1.37-fold; PSMB7: 1.25-fold (Figure 2(d)). In conclusion, the results so far underlined a general effect of EGb 761 on proteasome activity in the presence and absence of mutated htt proteins expression, potentially mediated by the expression of proteasome genes [29].

### 3.3. Modulating Effects of EGb 761 on Aggregation of PolyQ Proteins

Since an increased formation of polyQ aggregates was caused by the inhibition of the proteasome [32] and since enhancing global proteasome activity could prevent the formation of polyQ aggregates [33], we consequently tested whether the EGb 761-induced higher proteasome activity affects the actual formation of polyQ aggregates.

**Figure 3:**
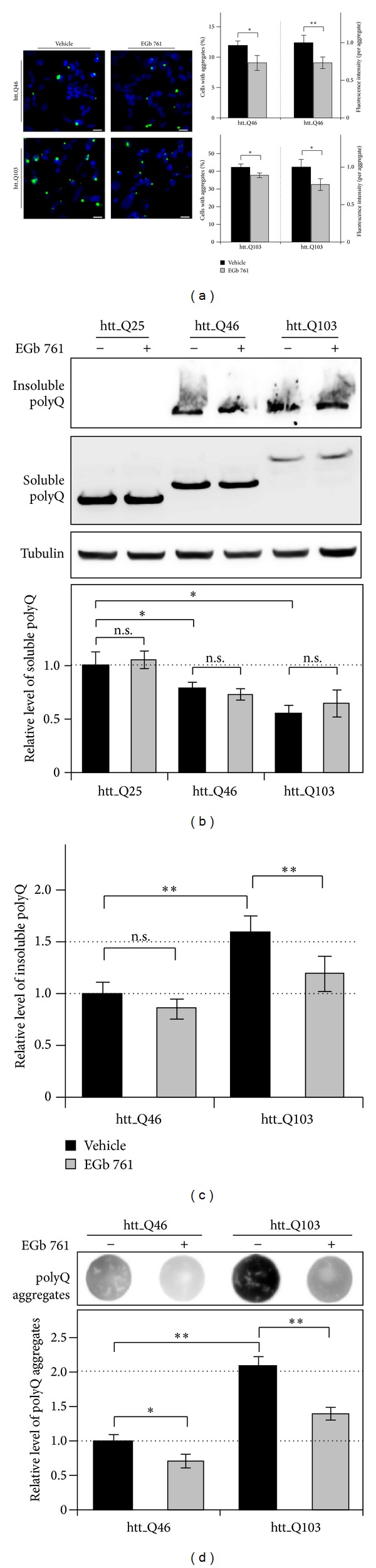
Modulating effects of EGb 761 on aggregation of polyQ proteins. (a–d) HEK293 cells expressing htt_Q46, htt_Q103, and (b) htt_Q25 were treated for 48 h with 150 *μ*g/mL EGb 761 or vehicle. (a) Fluorescence microscopy revealed significantly more polyQ aggregates in cells expressing htt_Q103 compared to htt_Q46. Note that most aggregates are formed as nuclear inclusions. Percentage of cells exhibiting fluorescent aggregates (%) was determined by plotting total amount of polyQ aggregates against total DAPI positive cells. Total cell and aggregate amount resulted from at least three independent experiments (more than 100 cells counted). Relative fluorescence intensity (a.u.) of aggregates was quantified by calculating aggregate mean intensity with aggregate area (pixel∗au). Representative pictures are shown. Scale bar: 20 *μ*m. (b) Cells expressing htt_Q25, htt_Q46, and htt_Q103 with EGb 761 or vehicle treatments were analyzed by immunoblotting. PolyQ proteins were detected with anti-eGFP antibody showing SDS-soluble protein bands. Cells expressing htt_Q46 and htt_Q103 showed aggregated SDS-insoluble proteins in the stacking gel (different exposure times were used). Densitometric values of soluble polyQ proteins from vehicle-treated htt_Q25-expressing cells were arbitrarily set to 1; *n* = 4. (c) Analysis of polyQ aggregation by using densitometric data of insoluble polyQ proteins from previous immunoblots (b). Values of vehicle-treated htt_Q46-expressing cells were arbitrarily set to 1; *n* = 4. (d) Whole cell extracts from cells expressing htt_Q46 and htt_Q103 with EGb 761 or vehicle treatment (samples from d) were subjected to a filter retardation assay. Detection of polyQ proteins trapped on nitrocellulose membrane revealed highly aggregated polyQ proteins in htt_Q103 compared to htt_Q46. Densitometric values of polyQ aggregates from vehicle-treated htt_Q46-expressing cells were arbitrarily set to 1; *n* = 4. (a–d) All values are reported as mean ± S.D. *P < 0.05 and **P < 0.01.

First, we analyzed HEK293 cells expressing htt_Q46 and htt_Q103 upon a 48 h treatment with EGb 761 using fluorescence microscopy and observed numerous cells with a distinctive accumulated GFP fluorescence indicating polyQ aggregates (Figure 3(a)). Interestingly, the EGb 761 treatment significantly decreased the number of cells bearing aggregates in cells expressing htt_Q46 to 0.75-fold and htt_Q103 to 0.9-fold (Figure 3(a)), accompanied by a significant decrease of aggregate fluorescence intensity (htt_Q46: 0.73-fold; htt_Q103: 0.77-fold to vehicle). These results indicate the presence of lower amounts of aggregated polyQ proteins as a result of a more sufficient degradation after EGb 761 treatment.

Next, we analyzed the direct effects of EGb 761 on polyQ protein aggregation in cells expressing htt_Q25, htt_Q46, and htt_Q103 (Figure 3(b)). Immunoblot analysis revealed decreasing levels of SDS-soluble polyQ proteins in correlation with polyQ protein length (htt_Q46: 0.78-fold, htt_Q103: 0.54-fold to htt_Q25), whereas the EGb 761 treatment showed no effect in all analyzed cells (Figure 3(b)). Additionally, cells expressing expanded polyQ proteins exhibited SDS-insoluble polyQ proteins correlating in their amount to the polyQ protein length, which were increased up to 1.59-fold in htt_Q103-expressing cells compared to htt_Q46 (Figures 3(b) and 3(c)). Thus, EGb 761 reduced the accumulation of insoluble polyQ proteins in htt_Q46 expressions to 0.86-fold and in htt_Q103 to 0.75-fold, compared to vehicle treatment (Figures 3(b) and 3(c)) indicating an improved degradation of polyQ proteins [34]. To confirm these data, we probed the same samples with the more quantitative filter retardation assay [20] (Figure 3(d)). The densitometric analysis revealed a significant increase of aggregated polyQ proteins in cells expressing htt_Q103 (2.1-fold) to htt_Q46-expressing cells (Figure 3(d)) as well as a significant decrease in aggregated polyQ proteins with the EGb 761 treatment in all cells compared to vehicle (htt_Q46: 0.71-fold; htt_Q103: 0.67-fold) (Figure 3(d)). As misfolded expanded polyQ proteins are barley degraded [35], nondegraded polyQ proteins accumulate into insoluble polyQ proteins and indicate the degradation efficiency of misfolded polyQ proteins [36]. Therefore, these findings suggest that EGb 761 improved the degradation of misfolded polyQ proteins that results in a reduced accumulation of insoluble polyQ proteins and decrease of polyQ aggregates in cells [37].

### 3.4. Modulating Effects of EGb 761 on Degradation of PolyQ Proteins

**Figure 4:**
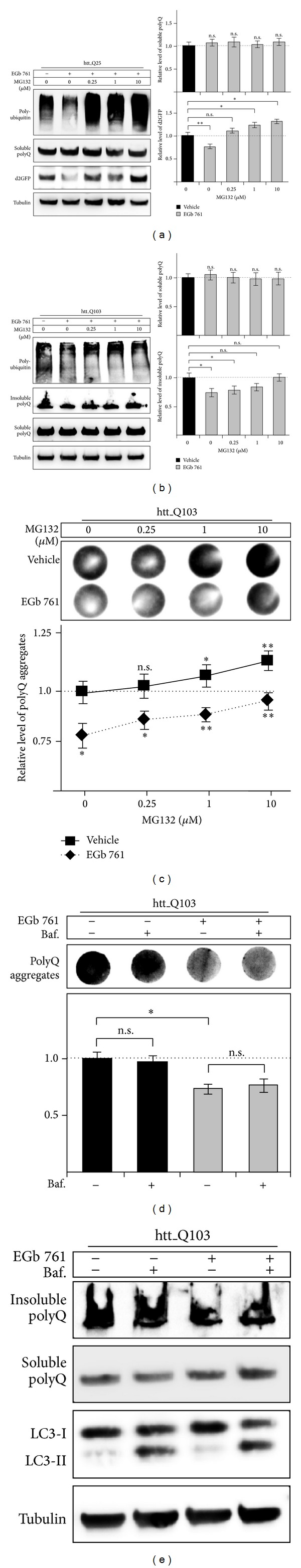
Effects of EGb 761 on degradation of polyQ proteins and polyQ aggregation. (a) d2GFP-HEK cells expressing htt_Q25 and (b–d) HEK293 cells expressing htt_Q103 were treated with EGb 761 or vehicle and subsequently chased with proteasome (MG132) or autophagy inhibitor (bafilomycin) to assess polyQ degradation and aggregation. (a) d2GFP-HEK cells expressing htt_Q25 were treated for 48 h with 150 *μ*g/mL EGb 761 or vehicle. Then, cells were incubated for 4 h with or without increasing concentrations of MG132. Protein levels of whole cell extracts were analyzed by immunoblotting to their corresponding antibodies. Protein levels of short-lived, unstable proteins (polyubiquitin, d2GFP) accumulated with proteasome inhibition while levels of long-lived polyQ proteins were not significantly altered. Values of d2GFP or polyQ protein of vehicle-treated cells without MG132 were arbitrarily set to 1; *n* = 4. (b-c) HEK293 cells expressing htt_Q103 were treated for 48 h with 150 *μ*g/mL EGb 761 or vehicle. Then, cells were subsequently incubated for 4 h with or without increasing concentrations of MG132. (b) Whole cell extracts were subjected to a filter retardation assay to assess polyQ aggregates, induced by pharmacologic proteasome inhibition. For the detection of aggregates of polyQ proteins trapped on nitrocellulose membrane an anti-eGFP antibody was used. Densitometric values of vehicle-treated cells without MG132 were arbitrarily set to 1; *n* = 5. (c) Protein levels of whole cell extracts (samples from b) were analyzed by immunoblotting to their corresponding antibodies. Protein levels of unstable, misfolded polyQ proteins accumulated with proteasome inhibition while levels of stable, soluble polyQ proteins were not altered. Values of soluble or insoluble polyQ proteins of vehicle-treated cells without MG132 were arbitrarily set to 1; *n* = 3. (d) HEK293 cells expressing htt_Q103 were treated for 48 h with 150 *μ*g/mL EGb 761 or vehicle. Then, cells were subsequently incubated for 3 h with or without 1 *μ*M of the lysosomal inhibitor bafilomycin A1 (denoted bafi.) to investigate the autophagic flux. Whole cell extracts were analyzed by immunoblotting or filter retardation assay. Cells treated with EGb 761 showed no significant alteration with bafilomycin treatment in protein levels of LC3-I to LC3-II, soluble and insoluble polyQ, indicating no direct effect on autophagy by EGb 761. Immunoblotting and filter retardation assay confirmed significant changes in aggregated polyQ proteins by EGb 761 from previous experiments (Figures 3(b)–3(d) and Figures 4(b)-4(c)). Densitometric values of vehicle-treated cells without bafilomycin were arbitrarily set to 1; *n* = 3. (a–d) All values are reported as mean ± S.D. *P < 0.05 and **P < 0.01.

To investigate the effects of EGb 761 on the degradation of polyQ proteins, we first analyzed d2GFP-HEK cells expressing htt_Q25 with or without an additional treatment with increasing concentrations of MG132. Immunoblot analysis confirmed the enhanced d2GFP degradation with EGb 761 as shown in Figure 2(b) by significantly reduced d2GFP levels (0.76-fold to vehicle) compared to vehicle treatment (Figure 4(a)), which were reversed by the pharmacologic proteasome inhibition up to 1.73-fold with 10 *μ*M MG132 (1.32-fold to vehicle) (Figure 4(a)). In contrast, protein levels of polyQ proteins (htt_Q25) were not significantly increased with the addition of MG132 (Figure 4(a)) suggesting a slower proteasomal degradation of these proteins. Moreover, EGb 761 treatment also considerably decreased levels of polyubiquitin proteins (denoted polyUb), which were reversed upon proteasome inhibition by MG132 (Figure 4(a)), furthermore confirming a more efficient degradation of ubiquinated proteins in EGb 761-treated cells [38]. Consequently, the EGb 761-induced enhanced proteasome activity was more effective in the degradation of unstable short-lived proteins (d2GFP, polyUb) than in soluble polyQ proteins due to their stability and prolonged half-life (Figure 4(a)).

Next, we investigated the molecular effects of EGb 761 on the proteasomal degradation of expanded polyQ proteins; we analyzed htt_Q103-expressing HEK293 cells with an addition of increasing concentrations of MG132. Immunoblot analysis confirmed that EGb 761 treatment decreases the amount of insoluble polyQ proteins (0.74-fold to vehicle) with no significant effect on soluble polyQ proteins as well as significantly lower levels of polyUb proteins in EGb 761-treated cells (Figure 4(b)). Moreover, addition of MG132 significantly increased the amount of insoluble polyQ proteins up to 1.36-fold (10 *μ*M MG132: 1.01-fold to vehicle) and no significant changes of soluble polyQ protein levels were found (Figure 4(b)). As expected, polyUb proteins accumulated in the presence of MG132, confirming the EGb 761-induced enhanced degradation of ubiquinated proteins (Figure 4(b)). Given the fact that pharmacologic proteasome inhibition increased levels of insoluble polyQ proteins compared to polyUb proteins, we conclude that insoluble polyQ proteins derive from nondegraded misfolded expanded polyQ proteins [20] and consist of unstable, short-lived proteins with a high proteasomal degradation frequency (Figure 4(b)). These results show that the observed effects of EGb 761 on reducing the amount of insoluble polyQ proteins were mediated by a more efficient proteasomal degradation of misfolded polyQ proteins [39]. Consequently, we investigated the degradation efficiency of misfolded polyQ proteins with EGb 761 treatment in comparison to vehicle treatment by using the formation of polyQ aggregates also in the presence of MG132 [40, 41]. We probed the samples from the previous experiments (Figure 4(b)) with the filter retardation assay. As expected, basic levels of polyQ aggregates were significantly lower in EGb 761-treated cells (0.78-fold to vehicle) compared to vehicle-treated cells (Figure 4(c)). Moreover, the amount of polyQ aggregates significantly increased in a dose-dependent manner upon pharmacologic proteasome inhibition, confirming that polyQ aggregates resulted from an inefficient proteasomal degradation. Hence, aggregate formation was increased with 10 *μ*M MG132 in EGb 761-treated cells up to 1.22-fold (0.95-fold to vehicle) and to a lower extent in vehicle-treated cells up to 1.15-fold (Figure 4(c)), indicating a more efficient polyQ protein degradation of EGb 761-treated cells [36]. Conclusively, these results confirmed that EGb 761 treatment induces a more efficient proteasomal degradation of misfolded expanded polyQ proteins, resulting in decreased amounts of polyQ aggregates (Figure 3).

Finally, as we have shown that EGb 761 reduces the amount of polyQ aggregates we tested the possible involvement of autophagy in the clearance of polyQ aggregates [42]. Therefore, we analyzed the general autophagosomal flux in HEK293 cells expressing htt_Q103 treated with EGb 761 or vehicle (Figures 4(d) and 4(e)), followed by a challenge with 1 *μ*M lysosome inhibitor bafilomycin A1. Analysis with the filter retardation assay revealed that basic levels of polyQ aggregates decreased in EGb 761-treated cells to 0.73-fold of vehicle-treated cells (Figure 4(d)). Interestingly, bafilomycin treatment did not alter the levels of polyQ aggregates in neither EGb 761-treated nor vehicle-treated cells (Figure 4(d)), indicating that autophagy had no effect on polyQ aggregates. Immunoblot analysis of the autophagosomal flux [43] confirmed that autophagy was active in EGb 761- and vehicle-treated cells indicated by the accumulation of autophagosome protein LC3-II in the presence of bafilomycin (Figure 4(e)). Nevertheless, LC3-II accumulation was not significantly altered with the EGb 761 treatment, suggesting that EGb 761 had no effect on autophagy. Besides that, inhibition of autophagy did not result in significant changes in soluble or insoluble polyQ protein levels (Figure 4(e)), confirming that autophagy was not contributing to the degradation of expanded polyQ proteins.

## 4. Discussion

Over the past decades, the* Ginkgo biloba* extract EGb 761 was used as a natural medication in a variety of neurodegenerative disorders, especially Alzheimer's disease (AD) and dementia [44]. Unfortunately, so far the reported results on the beneficial effects of EGb 761 in these disorders were rather inconsistent [45, 46]. Nevertheless, it is acknowledged that the multiple compounds found in EGb 761 may mediate pleiotropic effects on a vast number of proteins and genes suggesting a potential therapeutic and preventive use [2, 26].

Our results show that EGb 761 promotes basal proteasome activity (denoted PA) indicated by an increased proteasomal proteolytic activity and enhanced protein degradation, presumably through the observed EGb 761-mediated enhanced expression of catalytic proteasome genes. In support of such a mechanism are findings demonstrating an increased PA by the expression of proteasome genes through the activation of the NRF2-KEAP1 pathway [29, 30]. These studies reveal that NRF2 is the key transcriptional regulator required for the expression of proteasomal genes. Consistent with our own findings are studies showing the EGb761-induced transcription of phase 2 genes through the NRF2-KEAP1 pathway [10, 47].

Here, we used a fluorescent proteasome reporter protein (d2GFP) that provides an accurate tool to probe the proteasome status in living cells and animals [48]. Thus, the modulation of PA alters the proteasomal degradation of the reporter protein leading to changes of the fluorescence intensity [22]. We observed that incubation with EGb 761 decreases d2GFP fluorescence indicating an enhanced protein degradation that was confirmed by additional analyses with pharmacologic inhibitors of PA and protein biosynthesis. Furthermore, the assessment of PA with the proteasome-specific substrate SUC-LLVY-AMC [28] validates that EGb 761 modulates PA through an increase of proteasome enzymatic activity [49]. Nevertheless, besides the enhancement of the proteasome enzymatic activity, additional properties of EGb 761 could contribute to the sufficient protein removal [2].

Maintenance of cellular protein homeostasis is in part achieved by mechanisms of the protein quality control (PQC) [50], including two major mechanisms for protein disposal: the ubiquitin-proteasome-system (UPS) and autophagy [38]. The UPS and AP are pivotal for the distinctive clearance of misfolded and pathologically modified proteins. Otherwise aberrant proteins accumulate into protein aggregates, which is a common feature of several neurodegenerative diseases [11]. In HD, mutations in exon 1 of the* huntingtin* gene cause abnormal expansions of glutamine repeats in the N-terminus of the encoded protein (polyQ) [51]. Due to these unstable expansions some soluble expanded polyQ proteins would misfold into *β*-strand conformation, hereby becoming prone to self-interaction and aggregation [52, 53]. Furthermore, aggregation of these misfolded proteins mediates an impairment of the proteasomal degradation by the withdrawal of proteins from the UPS [54]. Therefore, clearance of expanded polyQ proteins through the UPS could be crucial to ameliorate pathological features of HD [33].

As we demonstrated an impact of EGb 761 on PA in native cells, we tested the effects of EGb 761 in cells expressing expanded polyQ proteins [54]. Our results confirmed a decrease of PA caused by the expression of expanded polyQ proteins [55] and a concomitant increase of 20S proteasome protein level. Because we did not find higher transcript levels of proteasome genes, we assumed the accumulation of proteasome proteins with polyQ aggregates and their inactivation [55]. Surprisingly, even under protein stress conditions EGb 761 treatment induced higher proteasome peptidase activity, increased d2GFP protein degradation, and elevated the transcription of proteasome genes, resulting in more proteasome proteins. These effects occurred in all cells despite their expression of polyQ proteins, indicating a ubiquitous impact of EGb 761 on PA that implicated an effect on polyQ protein aggregation. Indeed, it was demonstrated in models of cardiomyopathies that overexpression of proteasome genes increases the degradation of misfolded proteins and reduces protein aggregation [56]. Consequently, we showed that the expansion length of polyQ proteins correlated with lower amounts of SDS-soluble expanded polyQ proteins and the accumulation of SDS-insoluble expanded polyQ proteins, accompanied by high amounts of cells bearing polyQ aggregates [57], indicating an impaired clearance of polyQ proteins [34]. But cells treated with EGb 761 exhibited significantly lower levels of insoluble polyQ proteins and a reduced amount of cells bearing polyQ aggregates, indicating that EGb 761 modulates polyQ protein aggregation by a more efficient degradation of polyQ proteins through the enhancement of PA [57]. This assumption is supported by previous studies reporting the increased formation of polyQ aggregates with the impairment of PA and reduced degradation of polyQ proteins [14, 34]. Thus, upon pharmacological inhibition of the proteasome, we observed an increase of polyQ aggregates and insoluble polyQ proteins confirming the correlation between PA and polyQ protein aggregation [57]. Moreover, the effects of EGb 761 on reducing aggregation of polyQ proteins were abolished with the pharmacological inhibition of the proteasome showing that EGb 761 affects polyQ aggregation through the enhanced proteasomal degradation of polyQ proteins [40, 41]. Since we did not observe alterations in protein level of soluble polyQ proteins (htt_Q25 and htt_Q103) by the inhibition of proteasome activity, we suggested that polyglutamine tracts lack a strong proteasomal degradation signal affecting the protein turnover of soluble polyQ [36]. In contrast, we observed that destabilized, short-lived proteins and insoluble polyQ proteins accumulated very fast in the presence of a pharmacologic proteasome inhibitor and that insoluble polyQ proteins derive from improper cleared misfolded expanded polyQ proteins. Because misfolding of expanded polyQ proteins destabilizes the proteins, the misfolding serves as a “degradation” signal to direct misfolded polyQ proteins to proteasomal degradation [52]. More importantly, as unfolding of proteins is the basic requirement for the efficient degradation by the proteasome, misfolded polyQ proteins are reduced in their susceptibility to proteasomal degradation [58]. Interestingly, also other natural compounds such as the tender root of lei gong teng derived from traditional Chinese medicine approaches have been studied concerning potential effects on the activity of the cellular proteasome [59, 60].

Conclusively, these results showed that EGb 761 induced a faster degradation of misfolded polyQ proteins and alleviated formation of polyQ aggregates. Additionally, the proper clearance of polyQ proteins lowers the amount of polyQ aggregates causing less coaggregation of proteins from the UPS and results in a better protein homeostasis. In addition, we excluded ameliorating properties of EGb 761 on polyQ aggregation through higher rates of autophagy, as we demonstrated that inhibition of autophagy had no impact on polyQ aggregates. Since we did not observe alterations in protein level of SDS-soluble polyQ proteins (htt_Q25), EGb 761 was not inhibiting the expression of polyQ proteins* per se* or interfering with the polyQ aggregation nucleus by acting as a biochemical chaperone. Interestingly, consistent with our findings are results demonstrating that an enhanced degradation of expanded polyQ proteins results in lower levels of polyQ aggregates [39].

## 5. Conclusion

In summary, we found novel activities of EGb 761 on basal proteasome activity and protein degradation that is presumably mediated through the induced expression of proteasome genes.

We confirmed this effect of EGb 761 in cellular models of polyQ protein aggregation, hereby alleviating the polyQ-mediated proteasome impairment. In addition, EGb 761 reduced the aggregation of polyQ proteins through an enhanced degradation of misfolded polyQ proteins and ameliorated the amount of polyQ aggregates. These results indicate new properties of EGb 761 on polyQ protein aggregation through increasing the degradation of misfolded proteins. Future studies have to explore the therapeutic administration of EGb 761 as a potential treatment in the progression of polyQ protein associated Huntington's disease and other proteinopathies.
